# Endodontic Management of a Less Common Four-Rooted Maxillary Second Molar

**DOI:** 10.7759/cureus.84205

**Published:** 2025-05-15

**Authors:** Ammar Alammari, Faisal Alghamdi, Rayed Albeladi, Hassan Abed, Mohammed Yagmoor

**Affiliations:** 1 Department of Endodontics, Faculty of Dentistry, King Abdulaziz University, Jeddah, SAU; 2 Department of Oral Biology, Faculty of Dentistry, King Abdulaziz University, Jeddah, SAU; 3 Department of Restorative Dentistry, Faculty of Dentistry, King Abdulaziz University, Jeddah, SAU; 4 Department of Basic and Clinical Oral Sciences, Faculty of Dentistry, Umm Al-Qura University, Makkah, SAU; 5 Department of Endodontics, King Abdulaziz University Dental Hospital, Faculty of Dentistry, King Abdulaziz University, Jeddah, SAU

**Keywords:** endodontic therapy, four canals, maxillary molars, rare case, root canal therapy

## Abstract

This case report presents a less common anatomical variation in a maxillary second molar (MSM) characterized by four roots - two buccal and two palatal - and four distinct canals. The report emphasizes the significance of advanced diagnostic imaging and careful clinical evaluation in identifying and managing less common anatomical variations in maxillary second molars. Specifically, it presents the endodontic management of an MSM with two buccal and two separate palatal roots - detected using a cone beam computed tomography (CBCT) - underscoring the importance of comprehensive canal mapping and precise diagnosis in achieving treatment success. A 40-year-old male patient presented with intense pain in the upper right second molar, as reported by his general dentist. Clinical examination showed moderate pain on percussion and palpation. The radiological examination by CBCT scan revealed four separate roots and canals (two buccal and two palatal), with three canals filled with calcium hydroxide (Ca(OH)₂). Non-surgical root canal treatment was approached for the endodontic treatment. The nine-month follow-up showed resolution of all symptoms without pain on percussion and palpation. We conclude that a comprehensive understanding of the complex root canal morphology of MSMs is crucial for achieving successful endodontic outcomes. While common variations include three roots and three canals, less frequent configurations, such as a single root or four distinct roots with separate canals, emphasize the importance of comprehensive assessment. The prognosis currently demonstrates a higher success rate due to advancements in endodontic instruments and materials. Several factors play a role in success rate, such as the operator's experience, the number and location of canals, the treatment duration, and the utilization of tools like a microscope and CBCT, which can enhance the chances of achieving higher success rates in managing unusual variations of MSM.

## Introduction

Missing a canal because of individual anatomical differences can make it difficult to properly clean the entire tooth during endodontic treatment. This challenge often results in the failure of the procedure or ongoing symptoms after endodontic therapy. The success of non-surgical root canal therapies heavily depends on recognizing the complex variations in the tooth's internal structures. A thorough understanding of tooth anatomy is essential, especially in cases involving maxillary second molars (MSMs), where intricate structures demand careful imaging and skilled clinical techniques. These anatomical complexities pose significant hurdles, as the number of roots, their configurations, and accessory canals can differ greatly not only from person to person but even among similar teeth within the same population. Such variability complicates efforts to standardize treatment approaches, making it hard to achieve uniform results. These differences also influence how well the treatment works and how reproducible the outcomes are, especially when dealing with rare root forms like the four-rooted MSM described in this case. Therefore, detailed knowledge of tooth anatomy is fundamental to the success of root canal therapy, particularly because complex internal structures require precise imaging and expert handling [1].

Maxillary molars are known for their wide range of canal arrangements. Typically, MSMs have three roots: one located on the palate side, one on the mesiobuccal side, and another on the distobuccal side, with usually one canal in each root [2]. Failing to locate and properly treat all root canals is one of the main reasons for unsuccessful root canal procedures [3]. Nevertheless, the internal anatomy of these teeth can be unpredictable. Often, incomplete cleaning or filling of undetected canals is a key factor behind treatment failure [3]. Missed canals tend to occur because of their complex shapes, limited exploration, or the constraints of traditional two-dimensional (2D) X-rays. When untreated, these hidden canals can harbor bacteria, leading to ongoing inflammation around the tip of the root, reinfection, and ultimately, failure of the treatment. In endodontics, understanding the detailed root canal morphology is crucial because success hinges on thoroughly cleaning, shaping, and sealing all the existing pathways.

The presence of unusual or complex anatomical variations makes achieving this goal more difficult, especially when clinicians depend only on standard periapical radiographs. These 2D images often cannot reveal all the internal structures due to their projection limitations. Cone beam computed tomography (CBCT) provides a significant advantage in such situations by offering detailed three-dimensional views of the root canal system. Unlike traditional X-rays, CBCT helps visualize additional roots or canals that might be hidden or overlapped in 2D images. This improves diagnostic accuracy and supports better planning and execution of treatments [4].

Research has documented various rare configurations of root and canal structures in MSMs. Peikoff et al. [5] described six main types, with the most common being three separate roots and canals, accounting for around 56% of the cases. Following that are cases with three roots and four canals - specifically, two mesiobuccal canals - making up about 22.7%. Other variations include two roots with merging canals (9%), two separate roots (6.9%), a single root (3.1%), and less commonly, four roots with two palatal canals (1.4%) [5].

This case report aims to highlight the significance of advanced diagnostic imaging and careful clinical evaluation in identifying and managing less common anatomical variations in maxillary second molars. Specifically, it presents the endodontic management of an MSM with two buccal and two separate palatal roots - detected using CBCT - underscoring the importance of comprehensive canal mapping and precise diagnosis in achieving treatment success.

## Case presentation

Case history/examination

A 40-year-old fit and healthy male patient was referred to the endodontic clinics at the dental hospital (King Abdulaziz University, Jeddah, Saudi Arabia). The reason for the referral was to evaluate a previously initiated endodontic therapy for MSM (#17). The patient complained of intense and persistent pain. The patient mentioned undergoing a root canal treatment attempted by a general dentist one week prior. Upon clinical examination, extra-oral examination was non-significant, while intraoral examination revealed moderate pain to percussion and palpation, while periodontal examination showed healthy periodontal pockets and a mesio-occlusal glass ionomer temporary restoration.

**Figure 1:**
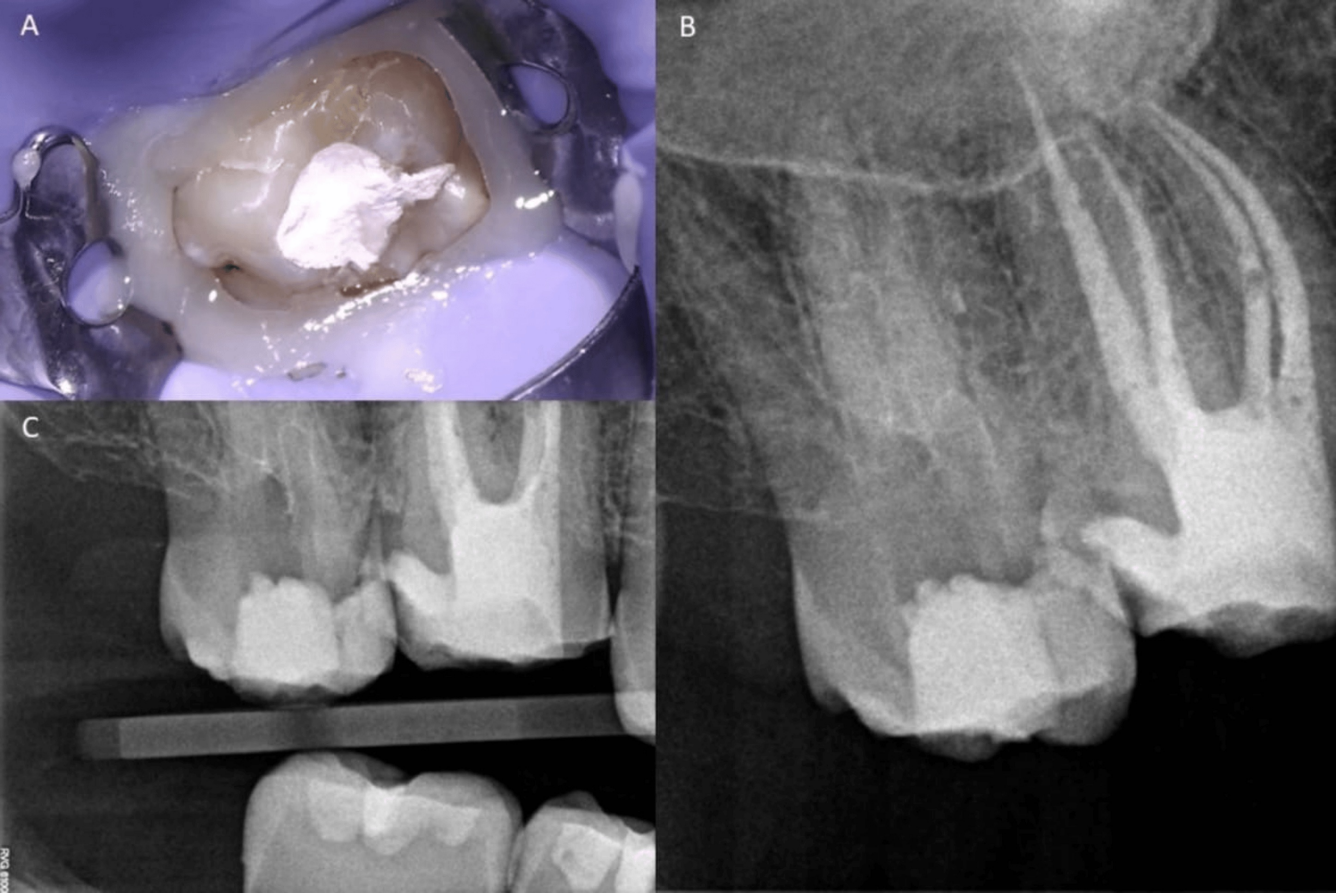
Preoperative clinical and radiographic images of tooth #17. (A): Preoperative clinical photograph showing temporary restoration on tooth #17 (Cavit and glass ionomer). (B): Preoperative periapical radiograph. (C): Preoperative bitewing radiograph

A cold test gave a negative response when compared to a tooth (#15) and the contralateral second molar (#27). Radiographic examination showed the presence of three roots and three canals filled with radiopaque materials and what appears to be a perforation in the mesial side of the tooth at the level of the crestal bone (tooth #17). The tooth had a mesio-occlusal glass ionomer temporary restoration (Figure 1). 

Methods (differential diagnosis, investigations, and treatment)

**Figure 2:**
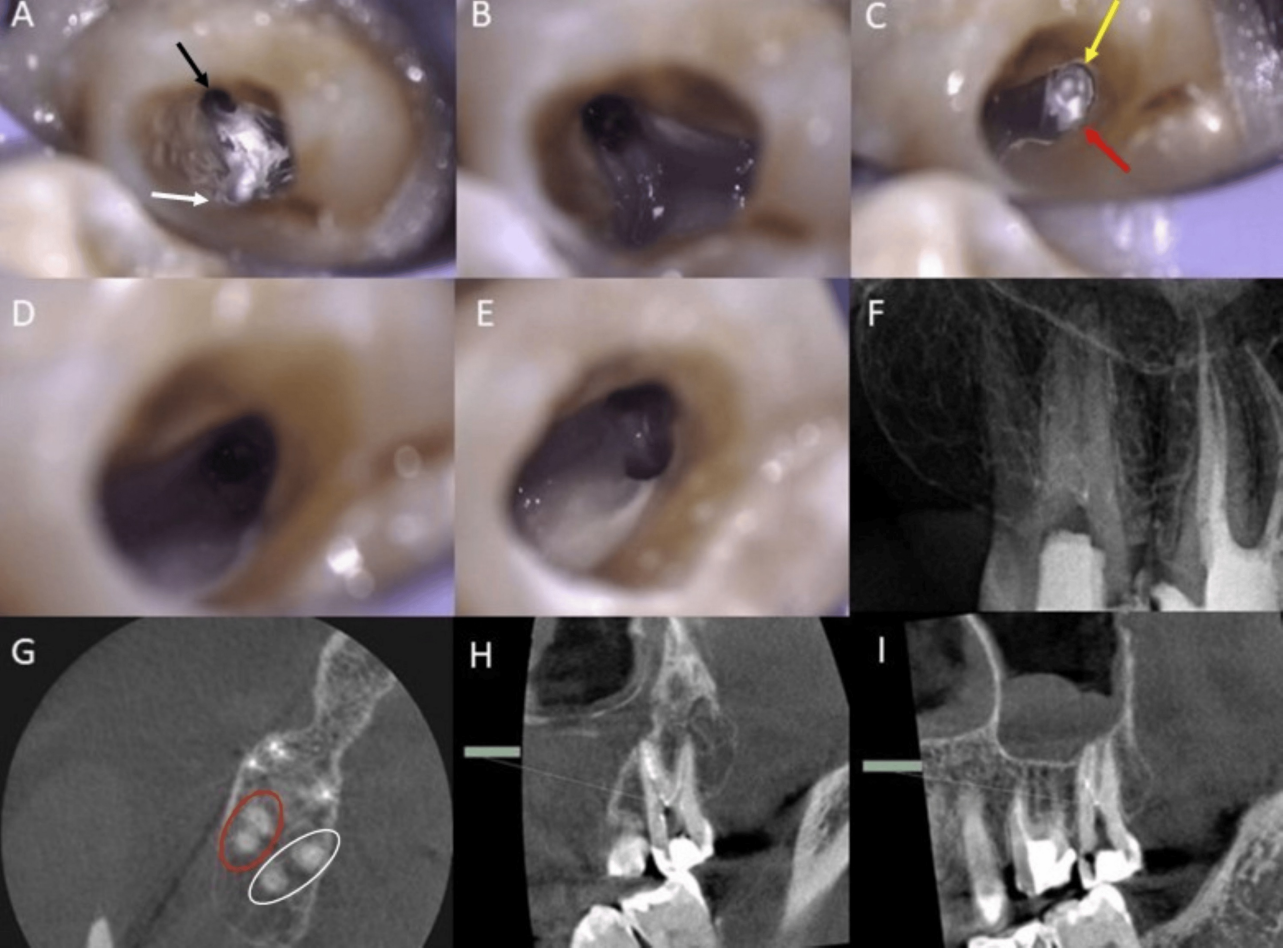
Intraoperative clinical and radiographic images of tooth #17 at the first visit (A) Photograph after accessing the tooth, showing two palatal canals filled with calcium hydroxide. Black arrow shows the mesiopalatal canal and white arrow shows the distopalatal canal. (B) After cleaning and shaping the palatal canals. (C) Mesiobuccal canal filled with calcium hydroxide. Yellow arrow shows the mesiobuccal canal, red arrow shows the location of the distobuccal canal. (D) After cleaning the missed distobuccal canal. (E) After cleaning and shaping the two buccal canals. (F) Periapical radiograph after calcium hydroxide application. (G) CBCT image (axial view) showing four separate roots. Red circle shows the orifices of the mesiobuccal and distobuccal canals align on the same line and are located buccally; the two buccal roots are parallel and close to each other, with no divergence. White circle shows the orifices of the mesiopalatal and distopalatal roots aligned on the same line and located palatally; the two palatal roots look widely divergent. (H) Sagittal CBCT view: the distance between the two palatal canals was 2.42 mm. (I) Sagittal CBCT view: the distance between the two buccal canals was 0.97 mm. CBCT: cone beam computed tomography.

Based on clinical and radiographic examinations, the tooth was diagnosed as previously initiated with symptomatic apical periodontitis. A pretreatment CBCT detected four separate roots and canals (two buccal and two palatal roots). After discussing various treatment options with the patient, local anesthesia was delivered using buccal infiltration of lidocaine with epinephrine (Dentsply Pharmaceutical, York, PA, USA) to start non-surgical root canal retreatment (NSRCRTx). After the placement of the rubber dam, the temporary restoration was removed, and the access cavity was modified. Three canals that were filled with Ca(OH)₂ were identified (mesiobuccal, mesiopalatal, and distopalatal) (Figure 2).

Careful examination of the pulpal chamber floor under a dental operating microscope (Carl Zeiss NTS Ltd, Oberkochen, Germany) showed the fourth distobuccal canal orifice in the mesiobuccal side of the pulpal floor distal to the mesiobuccal canal. The working length (WL) was determined using electronic apex locator (EAL) Root ZX II (J Morita, Tokyo, Japan) and confirmed radiographically. Further cleaning and shaping of all canals were done using EdgeFileX7 (EF) rotary files (Edge Endo, Albuquerque, NM, USA) (size 17, taper 0.4, then size 20, taper 0.4, size 25, taper 0.4, and size 30, taper 0.4). More apical preparation was required for both palatal canals up to size 35 (taper 0.4). Files were operated at 300 rpm/3 Ncm to the full WL. The canals were irrigated with sodium hypochlorite (5.5%). Ca(OH)₂ was placed in the canals followed by a temporary filling.

**Figure 3:**
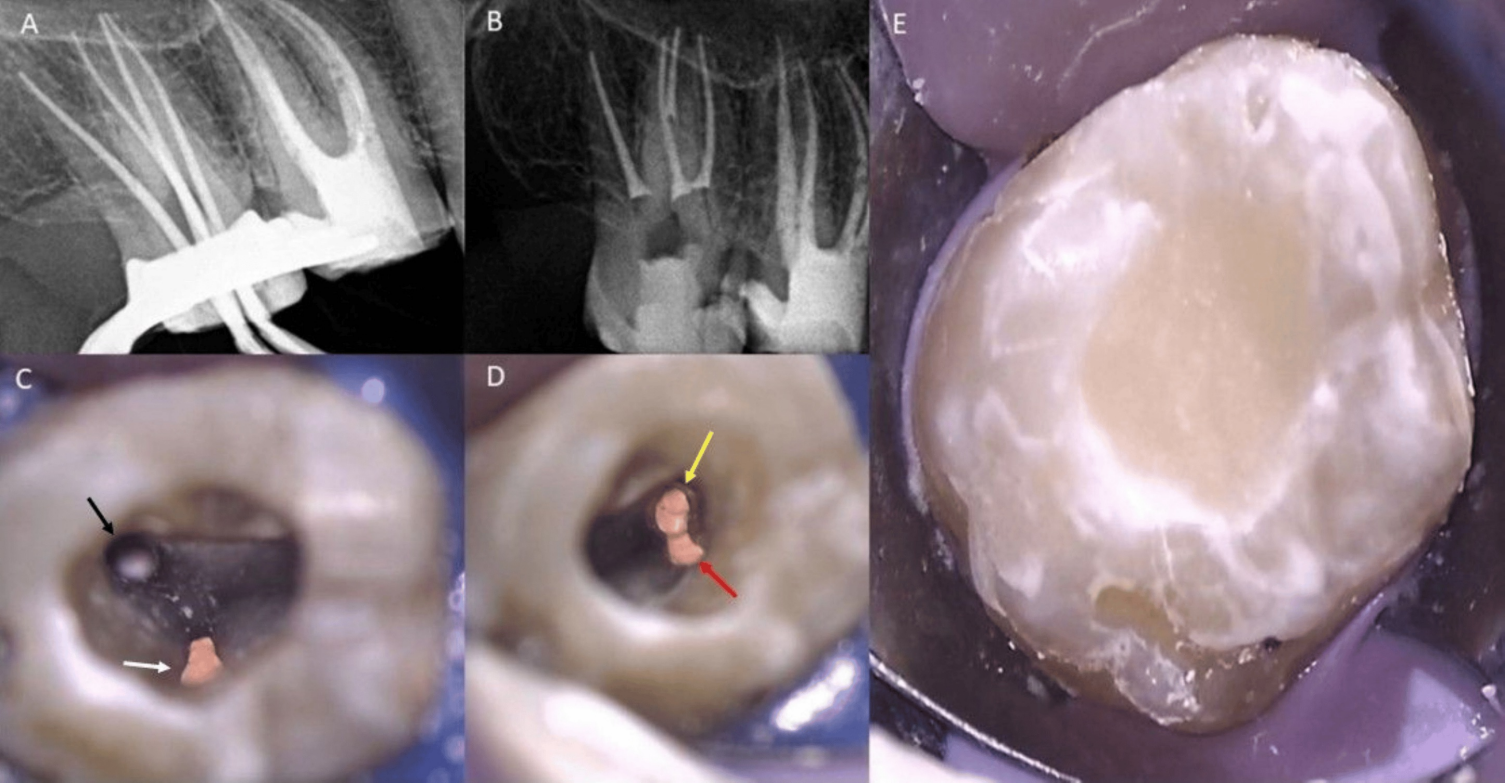
The clinical image of obturation and post-operative clinical and radiographical images of tooth #17 at the second visit. (A): Periapical radiograph showing master cone reaching full length in four canals. (B): Post-operative radiograph after obturation with temporary glass ionomer cement (GIC) filling. (C): Photograph of the two palatal canals after obturation. Black arrow shows the mesiopalatal canal with post space and white arrow shows the disto-palatal canal (D): Photograph of the two buccal canals after obturation. Yellow arrow shows the mesiobuccal canal, and red arrow shows the of the distobuccal canal. (E): Photograph after obturation and temporary GIC filling.

Two weeks later, no pain was reported. After root canal irrigation, master cones gutta-percha size #30/04 for the mesiobuccal and distobuccal canals and #35/04 for the two palatal canals were inserted to the full WL, and obturation was done using calcium silicate-based bioceramic sealer (CeraSeal, Meta Biomed, Cheongju-si, South Korea) in a sealer-based “single-cone” technique. Dental glass ionomer filling material was used post-NSRCT (Figure 3).

Conclusions and results (outcome and follow-up)

**Figure 4:**
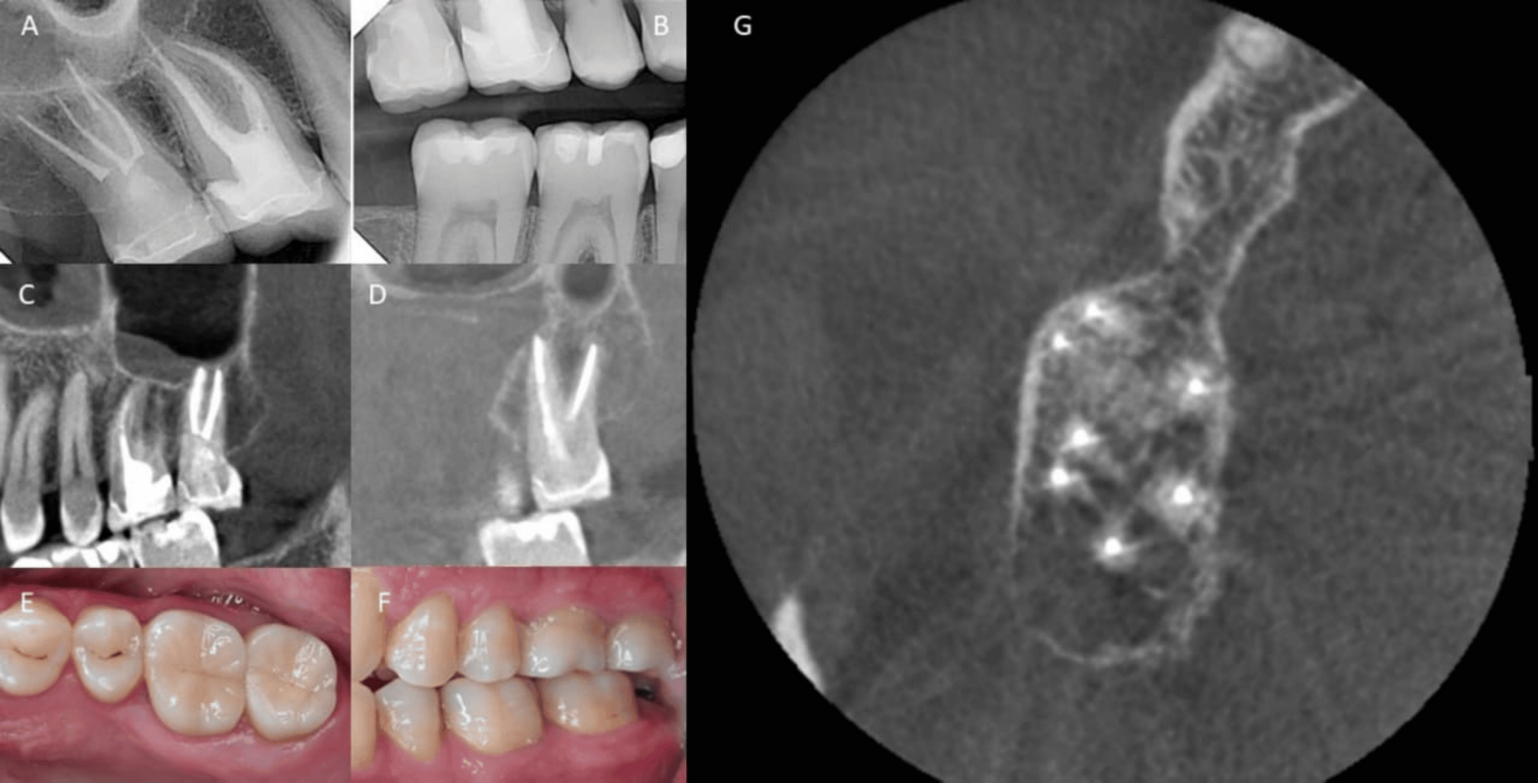
Clinical and radiographical images of follow-up visit after nine months for tooth #17. (A) Periapical radiographic image after a nine-month follow-up period. (B) Bitewing radiographic image after a nine-month follow-up period. (C) CBCT image of the sagittal view showing the two buccal canals after a nine-month follow-up period. (D) CBCT image of the sagittal view showing the two palatal canals after a nine-month follow-up period. (E) Clinical image showing the occlusal view for the tooth after crown placement after a nine-month follow-up period. (F) Clinical image showing the buccal view for the tooth after crown placement after a nine-month follow-up period. (G) CBCT image of the axial view showing four roots after a nine-month follow-up period.

Upon recall after nine months, the patient had undergone the placement of a new post and crown during this interval. Subsequently, all presenting symptoms had disappeared. Clinical assessment revealed the absence of pain on percussion and palpation, as well as no detectable mobility. The probing depth was within normal limits. Radiographic examination using CBCT demonstrated a state of normal periapical health without any signs of pathological findings (Figure 4).

The prognosis currently has a higher success rate with the development of advanced endodontic instruments and materials. Many factors can contribute to the success rate of an unusual variation of MSM cases, including the operator’s experience, the number and location of the canals, treatment duration, and the diagnostic tools used. With the use of advanced tools such as microscopes and CBCT, the chances of successful detection and treatment of complex canal anatomies increase. Studies have shown that the overall success rate of NSRCTs in maxillary second molars ranges from 72% to 95%, depending on canal complexity, instrumentation technique, and follow-up period [6, 7]. In cases involving atypical morphologies, such as additional canals or roots, missed anatomy has been cited as a major cause of failure [8]. Our case, involving a rare four-rooted MSM, illustrates the importance of comprehensive diagnosis and use of advanced imaging, which likely contributed to the favorable outcome observed. 

## Discussion

Differences in the root and canal morphology of MSMs have been reported in the literature. Research shows that while three-rooted configurations are most common in maxillary second molars, variations exist. Some studies report cases with only two roots, while others document up to five roots. This variation emphasizes the necessity of thorough diagnostic imaging, such as CBCT, to identify all anatomical complexities [9,10]. Understanding the wide range of root canal morphologies is crucial for endodontic success. Utilizing advanced imaging technologies, including CBCT, can help clinicians detect unusual root configurations, reducing the risk of missed canals and subsequent treatment failures [9,10].

Some authors reported differences in four-rooted MSM. Versiani et al. used micro-computed tomography to examine four-rooted extracted MSMs and found that all roots had a single canal. However, the mesiobuccal root exhibited two canals in 24% of the cases [11]. Zeng et al. [12] reported a case of an MSM featuring four roots and five canals. Sha et al. [13] presented a case report of a four-rooted MSM with five canals, where it had two palatal roots with two canals and two buccal roots with three canals, including a second mesiobuccal canal. Alkhalaghi and Fazlyab [14] documented a root canal treatment of an MSM, where two palatal roots were detected using CBCT. Another study documented a non-surgical root canal treatment of four-rooted MSM [15]. Gabriel Magnucki [16] also documented the management of a retreatment case of MSM with four roots and an untreated second mesiobuccal root. Peikoff et al. [5] found that approximately 1.4% of maxillary molars exhibited four distinct roots and four distinct canals, which included two palatal canals. 

Studies indicate that in the Saudi population, the majority of maxillary second molars have three roots. However, other studies have shown different numbers of roots, such as cases with two roots [17] or instances with four roots [18-21]. This prevalence underscores the importance for local practitioners to remain vigilant for atypical anatomical structures.

Christie et al. [22] described three classifications of maxillary molars with two palatal roots. In this case report, the maxillary second molar exhibited a Type I morphology, characterized by two widely divergent palatal roots (Figure 2 (H,I)). These teeth typically present with four distinct root apices. While in this case, we noted a predilection for this anomaly in maxillary molars, its identification is crucial for successful endodontic treatment, requiring a modified access cavity preparation to locate all the canal orifices.

Maxillary posterior teeth, especially second molars, are often in close proximity to the maxillary sinus floor [23, 24]. In a study focused on the Saudi population, Altaweel et al. identified the buccal roots of the maxillary second molars as being closest to the sinus floor [25]. According to Siqueira et al. (2021), apical periodontitis may expand and perforate the cortical floor and the periosteum of the sinus even if the root apex does not penetrate the sinus floor [26].

Maxillary sinusitis of endodontic origin (MSEO), as defined by the American Association of Endodontists (AAE) in 2018, is sinusitis caused by peri-radicular endodontic pathology. Apical periodontitis near the sinus mucosa can lead to periapical mucositis (PAM), radiographically visible as mucosal thickening or a dome-shaped soft tissue expansion adjacent to the affected root apex [27].

CBCT imaging is superior to conventional radiography in detecting odontogenic sources of sinusitis [28]. The primary goal in treating MSEO is the elimination of pathogens and debris from the infected root canal system [27]. Longhini and Ferguson found endoscopic sinus surgery (ESS) to be ineffective without prior dental intervention [29]. Yoo et al. reported that 67% of patients improved with medical and dental treatment alone, with only 33% requiring ESS. However, some authors still recommend ESS as the initial approach [30].

**Figure 5:**
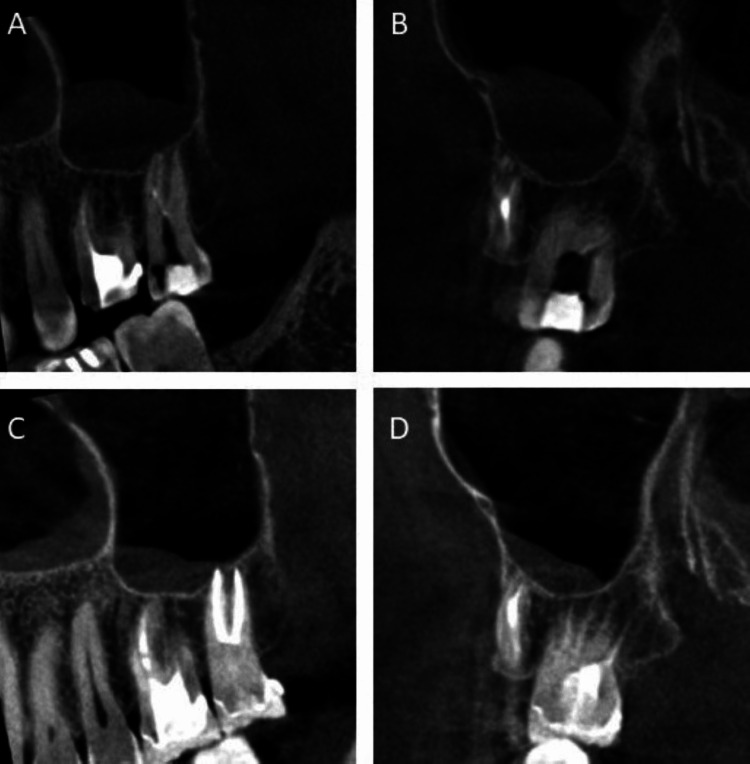
Preoperative vs. post-treatment CBCT images to show the healing of maxillary sinus at tooth #17. (A) Sagittal CBCT imaging provides a view of mucosal edema along the floor of the right maxillary sinus, potentially indicative of sinusitis. (B) A corresponding coronal CBCT view further illustrates the extent of the mucosal edema along the floor of the right maxillary sinus. (C) Sagittal CBCT view: Nine months postoperatively, a marked reduction in mucosal edema is evident, indicating successful healing. (D) Coronal CBCT view illustrates the resolution of mucosal edema in the right maxillary sinus. CBCT: cone beam computed tomography.

In this case, the initial presence of clear PAM appears on preoperative CBCT imaging as a mucosal thickening. A notable regression of mucosal thickening after the endodontic treatment can be seen in the nine-month follow-up, though some mucosal thickening still persisted (Figure 5) and this supports the literature advocating for a multidisciplinary approach.

Treating MSMs with complex root canal anatomy poses several technical challenges, including difficulty in locating and negotiating extra canals, and risks of perforation or incomplete obturation. To overcome these challenges, clinicians can benefit from advanced diagnostic tools such as CBCT, which provides three-dimensional imaging to clearly reveal complex root structures, and magnification via dental operating microscopes to improve visualization and precision during treatment. While non-surgical root canal treatments proved successful in the presented case, they may not always be the optimal treatment for more complex cases. In such situations, surgical endodontic procedures, such as apicoectomy and root-end resection, offer an alternative. These techniques aim to remove infected tissue at the root apex and seal the root, improving the chances of tooth retention [31]. Furthermore, extraction and implant placement are valuable options when the tooth cannot be preserved. Immediate implant placement following extraction has shown high success rates (93.3%), with a meta-analysis reporting survival rate after one year of follow-up was 96.6% for implants placed in molar extraction sockets [32].

A successful root canal treatment can be influenced by anatomical variability, which can be reflected either coronally or radicularly by an unusual number of extra canals, shape and curvatures, unusual root length and thickness, as well as complex internal canal structure like isthmuses and C-canals. This in return will add extra caution in regards to better cleaning and shaping, optimizing the instrumentation technique to avoid procedural errors like transportation, missing canals, perforation, or having the canals over-instrumented. Thus, in situations similar to this case, CBCT may add an advantage for anticipating anatomical complexity and modifying the techniques and instrument choice.

## Conclusions

Successful management of an unusual MSM with fourth canal in two roots is influenced by different factors as mentioned in this case. The variability of this type of tooth underscores the need for standardized guidelines to improve patient outcomes and contribute to best practices in such cases. For general dentists and endodontists treating complex cases, it is essential to employ comprehensive diagnostic methods like CBCT, refer early to specialists when necessary, and ensure clear communication with patients about treatment challenges and alternatives to help set realistic expectations.
